# MTL-DAS: Automatic Text Summarization for Domain Adaptation

**DOI:** 10.1155/2022/4851828

**Published:** 2022-06-15

**Authors:** Jiang Zhong, Zhiying Wang

**Affiliations:** Computer Science and Technology, Chongqing University, Chongqing 400044, China

## Abstract

Domain adaptation on text summarization task is always challenging, which is caused by the lack of annotated data in the target domain. Previous methodologies focused more on introducing knowledge in the target domain and shifted the model to the target domain. However, they mostly studied the adaptation to a single low-resource domain, which restricted practicality. In this paper, we propose MTL-DAS, a unified model for multidomain adaptive text summarization, which stands for Multitask Learning for Multidomain Adaptation Summarization model. Combined with BART, we investigate multitask learning method to enhance the generalization ability in multidomain. We adapt the ability of detect summary-worthy content from source domain and obtain the knowledge and generation style in target domains by text reconstruction task and text classification task. We carry out the domain adaptation ability experiment on AdaptSum dataset, which includes six domains in low-resource scenarios. The experiment shows the unified model not only outperforms separately trained models, but also is time-consuming and requires less computational resources.

## 1. Introduction 

Automatic text summarization [1, 2] is one of the important subtasks of natural language generation, which aims to compress long text into short text containing core information. There are two mainstream generation methods, namely, extractive summarization and abstractive summarization. The former [3] converts this task into a sentence sorting problem, and the latter [4] generates novel sentences by either rephrasing or using the new words. Abstractive summarization is always challenging as a generation task, which requires a large amount of human-annotated data. For low-resource areas that lack labelled data, domain adaptation automatic text summarization (DAS) [5, 6] task is researchable. DAS requires model to achieve adaptability on unknown or low-resource fields, without human intervention.

Hua and Wang [7] firstly studied the adaptability of neural text summarization models. They concluded that the model can learn to disclose summary-worthy content from the source domain data, which is transferable to a new domain. Recent works based on large-scale pretrained language models have proven to be predominate for DAS task, which are trained on massive unlabeled texts and huge parameters. Wang et al. [8] combined BERT [9] to explore the adaptive capabilities of text summarization models. Yu et al. [6] added a second stage of retraining based BART [10]. This work proposed a domain adaptability study of large-scale language model, and it created six different target domains in a low-resource scene. Whether introducing target domain knowledge based on the pretrained model [6, 8], or using target domain documents for secondary training [6], they all focused on the adaptation of a single field. However, an effective unified model for multidomain has not yet been proposed. In this paper, we propose MTL-DAS model with practical applicability, which can leverage available texts across multiple domains and enhance the model's generalization capabilities.

Previous studies on multidomain automatic text summarization are more reflected in the comparative analysis [9] and data generation methods [11]. Although these studies can support the adaptation and migration of multidomain text summarization, they cannot be applied. Multidomain adaptive summarization model should be guarantee of the relatively balanced performance between different source domains and target domains. It should have two properties: (1) the model should synergistically utilize the data of source domain and target domain, and (2) the model should be insensitive to the features that are interfering with the text summarization task. For such objective, we propose MTL-DAS model. We combine pretrained language model BART and investigate the multitask learning mechanism to build a unified model for multidomain adaptive summarization. To extract the shared knowledge across different domains and improve the model's domain adaptability, we leverage news domain as source domain. The numerous labelled data in the news domain make the model acquire the ability to detect summary-worthy key content, which can be adapted to new domains [7]. In addition to this, we use the AdaptSum dataset [6] as target domains, which include six domains in low-resource scenarios. The vocabulary, topics, and generation styles in six diverse domains are not alike; we introduce two auxiliary tasks, text reconstruction task and text classification task. The intuition behind this method is to obtain the knowledge and generation style in the target domains.

We experiment with various sharing modes and different weight coefficients settings. Due to unbalanced data volume in different domains, we test multiple sampling methods to reduce the distribution offset of domains. We finally obtain the Multitask Learning Model for Multidomain Adaptive Summarization and use the AdaptSum dataset proposed by Yu et al. [6] to carry out the domain adaptation ability experiment. Experiments show that the unified model has improved on part of low-source domains in AdaptSum dataset, and the practicability on multidomain is greatly enhanced. As a unified model, it not only outperforms separately trained models, but also is time-consuming and requires less computational resources.

Overall, our contributions are listed as follows:

- We propose Multitask Learning Model for Multidomain Adaptive Summarization (MTL-DAS). To the best of our knowledge, this is the first attempt to use the multitask learning in low-source multidomain adaptive automatic text summarization;
- Combined with BART, we investigate multitask learning method and experiment various sharing modes and weight coefficients to build an unified model for multi-domain. Multitask learning makes generated summaries aware of domain styles with adaptation of summary-worthy content ability. Experiments show that the unified model has improved on low-source domains in AdaptSum dataset, and the practicability on multidomain is greatly enhanced. We provide a paradigm for multidomain low-resource summarization tasks; it not only outperforms separately trained models, but also is time-consuming and requires less computational resources.

We introduce recent researches on multidomain and domain adaptation in Chapter 2, our model in Chapter 3, the experimental setup and analysis of results in Chapter 4, and Chapter 5 as a conclusion.

## 2. Related Work

### 2.1. Multidomain Research in Natural Language Processing

Researches on multiple fields in natural language processing can be classified into model-central, data-central, and hybrid methods. The model-central approach expands the feature space that the model can cover by changing the loss function, structure, or parameters. The data-central approach focuses on the data level, uses pseudolabels to construct data and bridges the distribution gap between domains [12, 13], optimizes domain adaptation through data selection methods [14], and enhances domain adaptation capabilities through pretrained methods [15, 16]. Our work is to expand the coverage area of the model through the processing of data in different domains and the collaborative training of multitask learning strategies without changing the original structure of the model. We provide a new idea and method for multidomain adaptive learning without changing model architecture.

### 2.2. Domain Adaptation in Automatic Text Summarization

The domain adaptation problem for natural language processing tasks has been studied extensively [17], but it has rarely been introduced into automatic text summarization tasks. Hua and Wang [7] were the first to study the adaptability of the neural text summarization model, and through experimental analysis, they concluded that the model can select key information from the source domain data. Gehrmann et al. [18] proposed a selective masking mechanism for generative text summarization and verified its adaptive ability on multidomain text data sets. Then, Wang et al. [8] conducted a comparative study on the domain migration problem of extractive automatic text summarization tasks. Rudar and Plank [12] studied the problem of cross-domain migration between two domains with completely different data distributions for generative automatic text summarization and gave cross-domain data generation methods. Yu et al. [6] added the second stage of retraining based BART, a pretrained language model for text summarization [10]. This work proposed a domain adaptability study of a large-scale language model, and it crossed six different target domains in a low-resource environment. Nevertheless, they are all different from the work of this article. Our work is multitask learning and collaborative training to enhance the generalization ability of the pretraining model. We showed the feasibility of multitask learning in domain adaptation and applied domain adaptation to multiple domains.

## 3. Model

In this section, we first present the overview structure of MTL-DAS that we propose for multidomain adaptation summarization. Then, we discuss how we set up multitask learning.

### 3.1. Overview Structure

**Figure 1:**
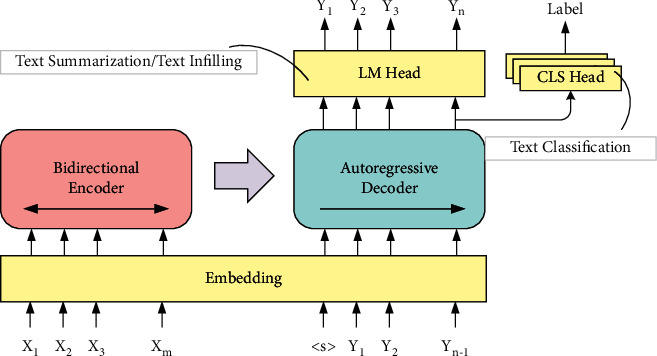
Overview structure of MTL-DAS.

We propose Multitask Learning Model for Multidomain Adaptive Summarization (MTL-DAS), and the architecture is shown in Figure 1. It incorporates BART as shared text encoding layers. The input (either a document with domain label or a document-summary pair) is first represented as a sequence of embedding vectors, one for each word. It captures the contextual information for each word. Our model learns abstractive summarization as a generation task using labelled data from both source and target domains. Domain style is learned by text reconstruction and text classification using unlabeled domain-related data from target domains. By learning abstractive summarization and domain style simultaneously through multitask learning, it is possible to generate a summary by considering the domain style of a given utterance. The model has several heads to be trained, including LM head for generation and CLS heads for classification. Given a domain-related sentence, the CLS head predicts its domain label.

BART [10] uses a standard Transformer-based neural machine translation architecture [19]. Transformer Encoder-Decoder architecture is mainly composed of multihead attention layers, norm layers, and feedforward layers. The formula of the self-attention mechanism is as follows:

(1) $$\begin{array}{c} \text{Attention}\left(Q,K,V\right)=\text{softmax}\left(\frac{QK^{T}}{\sqrt{d_{k}}}\right)V, \end{array}$$

where  *Q*, *K*, *V* represent the query matrix, the key matrix, and the value matrix, respectively. The dimension of the key and value matrices is *d*_*k*_. $1/\sqrt{d_{k}}$ is scale factor, which is applied to scale the weights of the Attention matrix obtained by dot product.

Multihead self-attention can be regarded as a stack of *h* heads; it uses a large matrix to accelerate the operation when calculating  *Q*, *K* and *V*.

(2) $$\begin{array}{c} \text{MultiHead}\left(Q,K,V\right)=\text{Concat}\left({\text{head}}_{1},\ldots ,{\text{head}}_{h}\right)W^{O}. \end{array}$$

In addition, Transformer Encoder-Decoder includes a feedforward layer composed of two layers of fully connected networks. The first layer of fully connected networks uses the ReLU activation function, and the second layer does not do nonlinear processing:

(3) $$\begin{array}{c} \text{FFN}\left(x\right)=\max\left(0,xW_{1}+b_{1}\right)W_{2}+b_{2}. \end{array}$$

Except for the basic network layers that constitute Transformer, the model uses Skip connection between layers to ensure the flow of input information before norm layer is performed.

According to the requirements of the text generation task, it adds several different noise functions based on token masking. We fine-tune it to the summarization task in the target domains. By multitask learning method, we adapt the ability of detecting key content that model learned from source domain and match the generation style of target domains.

### 3.2. Multitask Learning Setting

#### 3.2.1. Multitask Learning Based Domain Adaptation

Multitask learning method is the realization of “learning to learn,” which is using the useful information contained in the related tasks to provide a stronger inductive bias for the learning of the task concerned. When the main task is for less-labelled resource target domain, multitask learning method can boost the learning performance of it through the domain knowledge contained in the related task. Therefore, multitask learning is suitable for domain adaptation issues.

Under this premise, we investigate the multitask learning mechanism and set up abstractive text summarization as the main task, text reconstruction task, and text classification task as the auxiliary tasks. The multitask setup of our model is as follows. Aided by a huge amount of source domain labelled data, we train a model with strong identification of summary-worthy content. To match the generation style of target domains, we use text reconstruction task and text classification to maintain model's sensitivity to diverse domain styles. The details of tasks are as follows:

- Text summarization: abstractive summarization is the main task; we train this task on labelled data from source domain and target domains.
- Text reconstruction: a number of text spans are sampled and replaced with [MASK] tokens, and the lengths of spans are drawn from a Poisson distribution (*λ*=3). The model learns the number of masked tokens. This is one of the original pretraining objective functions, and the intuition behind this is to introduce the domain knowledge.
- Text classification: the input document is domain-related data from target domains. By recognizing original domains, the model is kept sensitive to the styles generated by different domains.

#### 3.2.2. Loss Function

MTL-DAS model combines three types of tasks: text summarization task, text classification task, and text reconstruction task. Let *θ* be the parameters of the model, and let **S**=[*s*_1_, *s*_2_,…, *s*_*T*_] denote a input sequence.

(i) Text summarization: the summary to **S** is defined as **X**=[*x*_1_, *x*_2_,…, *x*_*N*_]. The model infers an appropriate **X** from **S**. The loss ℒ_sum_ is calculated as the negative log-likelihood loss:
  (4) $$\begin{array}{c} {ℒ}_{\text{sum}}=-\sum_{n=1}^{N}\log\;p\left(x_{n}|S,x_{1},\ldots ,x_{n-1};\theta \right). \end{array}$$
After the model is trained, the sequence $\hat{X}=\left[{\hat{x}}_{1},{\hat{x}}_{2},\ldots ,{\hat{x}}_{N}\right]$ is generally generated by the following greedy search method. The output with the highest probability is selected at each time step, where ${\hat{x}}_{N}$ represents the *N* − *th* step output generated:
  (5) $$\begin{array}{c} {\hat{x}}_{N}=\underset{x_{N}}{\arg\max}p\left({\hat{x}}_{N}|{\hat{x}}_{1},\ldots {\hat{x}}_{N-1},S\right). \end{array}$$
(ii) Text reconstruction: we use its original pretraining objective function, corrupt documents, and then optimize a reconstruction loss, which is the cross-entropy between the decoder's output and the original document.
  (6) $$\begin{array}{c} {ℒ}_{\text{recon}}=-\sum_{t=1}^{T}\log\;p\left(s_{t}|\hat{S},S;\theta \right). \end{array}$$
Where $\hat{S}$ is perturbed text from **S**.
(iii) Text classification: given a domain-related text **S**, the model labels it using domain labels. The intuition behind this task is to help the model recognize different domains.

When dealing with K-classification problems with neural networks, the output layer uses softmax function as the activation function, which is

(7) $$\begin{array}{c} y_{k}\left(S,\theta \right)=\frac{\exp\left(a_{k}\left(S,\theta \right)\right)}{\sum_{j}^{K}\exp\left(a_{j}\left(S,\theta \right)\right)},\;\left(1\leq k\leq K\right), \end{array}$$

*y*_*k*_(**S**, *θ*) shows that the probability of **S** belongs to *k*. So, for any sample {**S**, *l*}, *l* is the correct label of **S**:

(8) $$\begin{array}{c} p\left(l|S;\theta \right)=\prod_{k=1}^{K}y_{k}{\left(S,\theta \right)}^{l_{k}}. \end{array}$$

The negative log-likelihood loss is used for the classification loss ℒ_cls_,

(9) $$\begin{array}{c} {ℒ}_{\text{cls}}=-\log\;p\left(l|S;\theta \right). \end{array}$$

#### 3.2.3. Loss Weighting

Assuming that the loss function ℒ_*i*_ of each task *i* has a weight coefficient *ω*_*i*_, the total loss is the weighted sum of all the task losses:

(10) $$\begin{array}{c} {ℒ}_{\text{MTL}}=\sum_{i}\omega_{i}\cdot {ℒ}_{i}. \end{array}$$

When the model starts training, the total loss is minimized by gradient descent, and the weight shared by model is updated by following formula:

(11) $$\begin{array}{c} W_{sh}=W_{sh}-T\sum_{i}\omega_{i}\frac{\partial {ℒ}_{i}}{\partial W_{sh}}. \end{array}$$

The value of *ω*_*i*_ determine the impact of each task on the shared weight update. Based on previous experience, the following three training target weight coefficients are selected:

- Empirical value: according to the settings of previous experience, we set the weight coefficient of the summarization task to 1, and the weight coefficient of the language model task to 0.02;
- Inversed ratio (by data size): reverse the size of dataset for different tasks as the weight coefficient (note that this method uses the size of the original training data);
- Dynamic weight averaging: according to the loss function ℒ_*i*_ obtained by task training, *ω*_*i*_ is automatically calculated.

Specifically, in Dynamic Weight Averaging [20], the calculation formula for the weight coefficient of the *i*-th task at the *i* th step is

(12) $$\begin{array}{c} \omega_{i}\left(t\right)=N\frac{\exp\left(r_{i}\left(t-1\right)/T\right)}{\sum_{n}\exp\left(r_{i}\left(t-1\right)/T\right)}. \end{array}$$

Among them, *N* is the total number of tasks, *T* is used to control the softmax operation, and the scalar *r*_*n*_(*·*) shows the relative decrease rate of the loss function for each task:

(13) $$\begin{array}{c} r_{n}\left(t-1\right)=\frac{{ℒ}_{n}\left(t-1\right)}{{ℒ}_{n}\left(t-2\right)}. \end{array}$$

When a task has a slower loss reduction rate compared with other tasks, the weight coefficient of the task will increase. Therefore, DWA can calculate the dynamic weight coefficient through the value of the loss function of each task.

## 4. Experiment

### 4.1. Dataset

We use XSum dataset [21] as source domain. Compared to other news domain datasets, it tends to generate new words rather than copying words from input sentences. We leverage the AdaptSum dataset proposed by Yu et al. [6] to carry out the domain adaptation ability experiment. AdaptSum provides an open evaluation benchmark for the abstractive text summarization model. It contains six different target domains and their corresponding unlabeled domain text. The target domains are as follows:

- Dialog: dialogue data, proposed by Gliwa et al. [22], is a manually annotated chat dialogue text dataset for abstractive summarization. The corresponding unlabeled dialogue data consists of Reddit conversations, personalized dialogs [23], empathetic dialogs [24], and Wizard of Wikipedia dialogs [25] data sets.
- E-mail: the e-mail summary dataset is proposed by Zhang and Tetreault [26], which is composed of e-mail and question pairs, including personal and business correspondence emails.
- Movie Review: movie review summary dataset is proposed by Wang and Ling [27].
- Debate: the debate summary dataset is proposed by Wang and Ling [27], which contains the thesis and the argument pairs. The corresponding unlabeled data comes from Ajjour et al. [28].
- Social Media: Kim et al. [29] obtained a summary dataset from Reddit TIFU, and the summary is the title of the blog post.
- Science: the abstractive text summary dataset for computational linguistics papers is proposed by Yasunaga et al. [30].

For Dialog and e-mail domain datasets, we follow the processing method of Yu et al. [6] and adopt its standard segmentation scheme. For the Movie Review, Debate, Social Media, and Science domains, since the original source of these datasets did not give a division method, a random division method is adopted, with a division ratio of 8 : 1 : 1.

Due to the small amount of data in Movie Review and Debate fields, the maximum number of training samples is set to 300. However, there are more data in Dialog, e-mail, Social Media, and Science fields, so 300 samples are randomly selected from each field to construct its corresponding low-resource dataset. In addition, we follow the maximum length limit of BART and process documents in all fields into a length of 1024 tokens.

**Figure 2:**
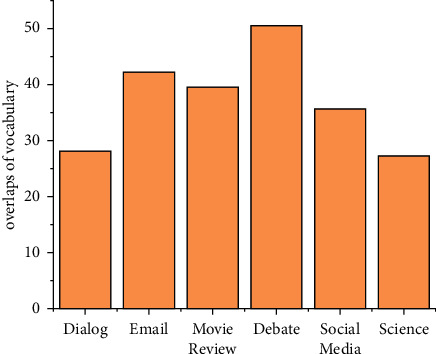
Vocabulary overlaps of the summarization validation set.

Figure 2 shows the vocabulary overlaps of the summarization validation set between target domains and source domain (Xsum). The figure illustrates that the overlaps between domains are comparably small, and the chosen domains are diverse, which brings huge challenges to domain adaptation task.

**Figure 3:**
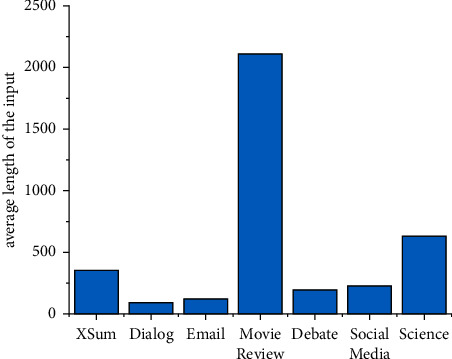
Averaged length of the input documents for the source and target domains.

As shown in Figure 3, we also statistically compare the average length of annotated data input in seven datasets and find that the length of movie review dataset and science dataset is particularly long.

### 4.2. Data Sampling Processing Method

In order to balance the distribution deviation, which is caused by the data volume difference between various domains for training, a reasonable sampling method needs to be adopted. To guarantee that each sample in the original training data is sent to the model at least once, a full sampling method is used in our model.

The following are three different sampling methods used in our training model:

- Random sampling. Without any processing progress, the original training data is scrambled and randomly obtained a data sample of batch size. The constituted batch is sent to train the model.
- Data enhanced sampling. Considering that the amount of data in various domains and tasks is quite different, the smaller data is enhanced first, and we adopt backtranslation method to keep the data in each domain at the same order of magnitude. Then, after data enhancement, random sampling is executed.
- Tasks sequential sampling. After data enhancement using back translation method, the task sequence is fixed. Samples in each field are sampled in turn for filling until the batch size is satisfied. (Keep the number of data in each domain in the batch sent to the model training the same.)

The best sampling method will be selected to report on the training result, and the impact of different sampling methods will be detailed in the experimental analysis.

### 4.3. Multitask Sharing Mode

In the process of multitask learning, the cooptimization of the model is usually achieved through the hidden layer parameters sharing.

The hard sharing mode shares all hidden layers among all tasks and differs only in the final single or multiple output layers (usually fully connected layers). The soft sharing mode retains separate model parameters for each task but maintains the similarity of parameters between multiple tasks by regularizing the distance between model parameters. We adopt L2 regularization as the distance regularization function of the soft sharing mode.

The same as data sampling method processing in the previous section, the model is trained on both hard sharing and soft sharing modes, and we select the best sharing mode. It should be noted that when the soft sharing mode is adopted, due to the limitation of training equipment, the five language model training tasks mentioned before need to be regarded as one task. This means that all tasks of the language model still adopt the hard sharing mode.

### 4.4. Baseline

Since we use BART as the backbone and adopt the multitask learning method to obtain the MTL-DAS model, the relevant pretrained models are selected as baseline methods for comparison:

- BART fine-tuning [10] performs supervised fine-tuning of BART parameters for the summarization task in each domain.
- AdaptSum [6] is the basis of our work and the proposer of the dataset. After the first stage of fine-tuning in the six low-resource domains, three second-stage pretraining ones were added. They use source domain documents (SDPT), target domain unlabeled documents (DAPT), and summarization task-specific target domain documents (TAPT), respectively, for second-stage pretraining.

### 4.5. Experiment Setting

We use BART-Base as the basic component of model realization in all experiments. Affected by the memory of the training device, the minibatch size is set to 4 in the model training, and the gradient accumulation is set to update every 10 iterations. The model uses the Adam optimizer, and the momentum parameter settings are *β*_1_=0.9, *β*_2_=0.998. The learning rate decay mode is Noam, and the number of warming up steps is set to 1000.

In the decoding stage, the model uses beam search to enhance the coherence of the generated text, and beam size is set to 4. The sequence end identifier EOS or the maximum generation length of 256 is used as the end condition in the decoding process.

### 4.6. Result

#### 4.6.1. Evaluation Method

Following the previous work [6], we use ROUGE to be the main evaluation indicator. ROUGE measures the quality of the abstract by calculating the overlap of the token between the generated abstract and the artificial abstract, including unigram, bigram, trigram, and the longest common subsequence (LCS). Since diverse data from six different fields are used for training and prediction, in order to intuitively reflect the efficacy of the model, we use ROUGE-1 (Unigram) to evaluate the model reasoning accuracy.

We use the official ROUGE evaluation script (v1.5.5 version)^1^ implemented by Perl to evaluate the generated abstract.

#### 4.6.2. Performance Evaluation Results

**Figure 4:**
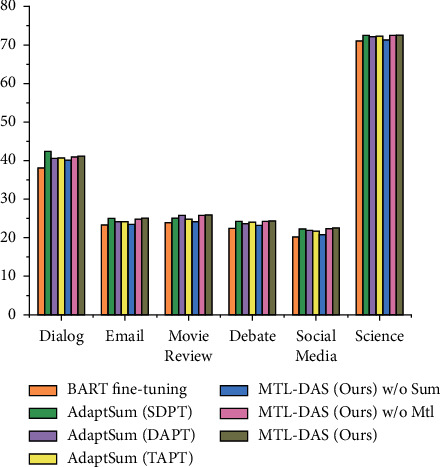
Performance evaluation results.

We show the results of baseline models in Figure 4. Although MTL-DAS does not overall exceed AdaptSum in six domains, its results are still competitive. As a whole model, its practicality on multidomain is better than that of AdaptSum trained in a single domain. The experimental results show that MTL-DAS has an overall better inference accuracy in six fields than the fine-tuning of BART. Even without using labelled data from target domains (w/o Sum), only using unlabeled domain text for training, and directly testing in the field to verify its field adaptation ability, the reasoning accuracy of most fields still exceeds BART. This result verifies the effectiveness of the multitask learning strategy we investigated. Without the assistance of multitask learning (w/o Mtl), through simultaneous training of the source domain data and a small amount of labelled data in the target domain, a comprehensive improvement result can still be obtained. This shows that the ability of capturing key content can be transferred. However, compared with SDPT, the effect is reduced, which indicates that content in different fields can still cause confusion. As a unified Multitask Learning Model for Multidomain Adaptive Summarization (MTL-DAS), it shows the strong ability of adaptation to multiple low-source target domains.

The specific values are shown in Table 1. The best experimental result is shown in bold, and the second best result is underlined. Although the improvement of MTL-DAS in various fields is not a leap, it is time-consuming and requires less computational resources.

### 4.7. Ablation Experiment Analysis

#### 4.7.1. The Impact of Different Sampling Methods

**Figure 5:**
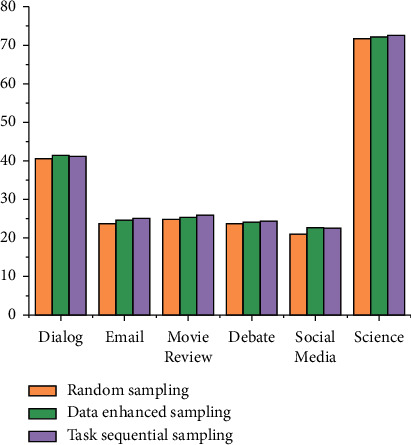
The impact of different sampling methods.

As shown in Figure 5, compared with random sampling, data enhanced sampling and tasks sequential sampling both have data enhancement, so their performance is improved.

The specific values are shown in Table 2. It can be seen from the table that the effect of data enhanced sampling in Dialog and Social Media is more substantial than tasks sequential sampling. According to data analysis and observation, it can be found that the amount of data in the two fields is significantly higher than that in other fields. The task sequential sampling will affect the composition of minibatch during model training, which makes large-scale corpus generate sample errors due to sorting. Due to the relatively effect in several other fields, task sequential sampling was finally selected as the final sampling method of MTL-DAS.

#### 4.7.2. The Impact of Different Sharing Modes

**Figure 6:**
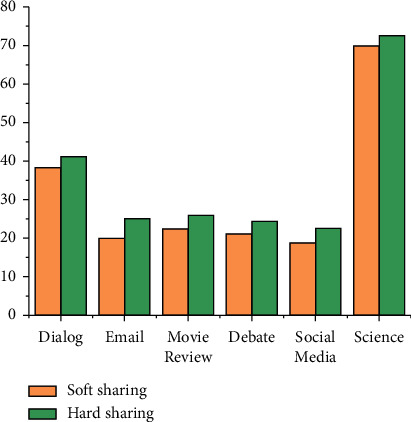
The impact of different sharing modes.

Using the two different weight sharing modes of multitask learning model, the obtained performance presents a relatively large difference. As shown in Figure 6, the hard sharing mode is 3%–5% higher on the ROUGE-1 score in all domains. Considering the large number of parameters of the BART model, it is difficult to use the soft sharing mode to effectively update the weight of the corresponding model for each task, so the performance is poor. The specific values are shown in Table 3.

#### 4.7.3. The Impact of Different Loss Weight Coefficients

**Figure 7:**
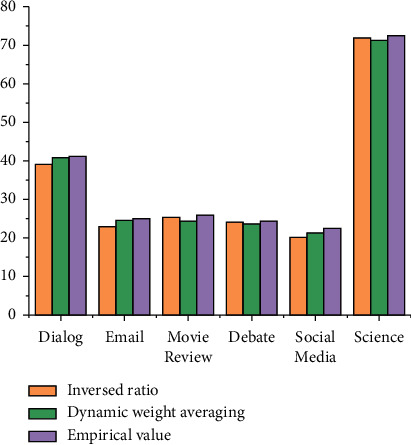
The impact of different loss weight coefficients.

As an important factor affecting the final multitask learning loss function, the loss of weight coefficient needs to achieve the best possible effect.  Figure 7 shows the experimental results. The data shows that the empirical value strategy has the best effect, and the inversed ratio (by data size) strategy performs better on Movie Review, Debate, and Science areas with fewer data. However, due to the low weights set for several other areas, its ROUGE score did not rise but fell. According to the decline of loss, the dynamic weight averaging strategy can automatically adjust the weight coefficient. But some areas may have slight changes in loss after training convergence, gradually reducing its impact on the overall loss, making the model fall into an unoptimized point. Therefore, the final model still chooses empirical values and sets static loss weight coefficients. The specific values are shown in Table 4.

## 5. Conclusion

We propose MTL-DAS model, a unified model for multidomain adaptive text summarization. Combined with pretrained language model BART, MTL-DAS model enhances the generalization ability in multidomain through multitask learning. To extract the shared knowledge across different domains and improve the model's domain adaptability, we leverage source (news) domain to acquire the ability to detect summary-worthy key content, which can be adapted to new domains. To obtain the knowledge and generation style in the target domains, we introduce two auxiliary tasks, text reconstruction task and text classification task. We carry out the domain adaptation ability experiment on AdaptSum dataset, which includes six domains in low-resource scenarios. The experiment shows that the unified model not only outperforms separately trained models, but also is time-consuming and requires less computational resources. In the future, we will study how to extract domain features from a small number of samples in the absence of unlabeled data.

## Figures and Tables

**Table 1:** ROUGE score for six target domain datasets.

Model	Dialog	E-mail	Movie review	Debate	Social media	Science
BART fine-tuning	38.14	23.27	23.89	22.38	20.17	71.04
Adapt sum (SDPT)	**42.33**	*24.97*	25.06	*24.17*	*22.25*	*72.49*
Adapt sum (DAPT)	40.58	24.15	*25.77*	23.64	21.83	72.15
Adapt sum (TAPT)	40.69	24.12	24.84	24.01	21.65	72.28
MTL-DAS (ours) w/o sum	40.09	23.46	24.16	23.14	20.77	71.33
MTL-DAS (ours) w/o mtl	40.95	24.84	25.74	24.21	22.32	72.47
MTL-DAS (ours)	*41.17*	**25.03**	**25.94**	**24.37**	**22.54**	**72.50**

**Table 2:** The result of ablation experiments for different data sampling methods.

Sampling methods	Dialog	E-mail	Movie review	Debate	Social media	Science
Random sampling	40.55	23.70	24.85	23.68	20.99	71.69
Data enhanced sampling	41.37	24.59	25.27	24.08	22.67	72.10
Task sequential sampling	41.17	25.03	25.94	24.37	22.54	72.50

**Table 3:** The result of ablation experiments for different sharing modes.

Sharing modes	Dialog	E-mail	Movie review	Debate	Social media	Science
Soft sharing	38.27	19.98	22.36	21.10	18.75	69.90
Hard sharing	41.17	25.03	25.94	24.37	22.54	72.50

**Table 4:** The result of ablation experiments for different loss weight.

Loss weight	Dialog	E-mail	Movie review	Debate	Social media	Science
Inversed ratio	39.17	22.94	25.30	24.12	20.19	71.96
Dynamic weight averaging	40.87	24.58	24.36	23.74	21.35	71.33
Empirical value	41.17	25.03	25.94	24.37	22.54	72.50

## Data Availability

The labelled dataset used to support the findings of this study is available from the corresponding author upon request.
